# Trigeminal Neuralgia as an Initial Presentation of Systemic Autoimmune Diseases: A Case Series

**DOI:** 10.31138/mjr.33.3.333

**Published:** 2022-09-30

**Authors:** Debashis Maikap, Prasanta Padhan

**Affiliations:** Department of Clinical Immunology and Rheumatology, Kalinga Institute of Medical Sciences, KIIT University, Bhubaneswar, Odisha, India

**Keywords:** connective tissue disease, trigeminal neuralgia, MCTD, Sjögren’s syndrome, systemic sclerosis, autoimmune disease

## Abstract

**Background:**

Trigeminal neuralgia, also known as tic doloureaux, refers to a sudden onset of unilateral or bilateral facial numbness with or without pain or paraesthesia. Trigeminal neuralgia is rare in connective tissue diseases (CTD); however, it is the most common neurologic manifestation of mixed connective tissue (MCTD) and maybe the only presenting symptom in various CTDs.

**Patients and Methods:**

Here, we describe a series of four cases of various autoimmune connective tissue diseases where trigeminal neuralgia was the presenting complaint. The first 2 cases were MCTD patients, and the 3rd case was a patient with diffuse cutaneous systemic sclerosis (SSc) and the 4th case had overlap syndrome (primary Sjogren’s syndrome with SSc). The relevant literature describing trigeminal neuralgia in CTD was reviewed. The authors performed a systematic search of patients with Trigeminal neuralgia and Connective tissue diseases in PubMed, Scopus from January 1970 until July 2022.

**Results:**

All our cases had trigeminal neuralgia as presenting symptom which suggests that trigeminal neuralgia may be one of the presenting symptoms of several systemic autoimmune diseases that often cause a significant delay in diagnosis and treatment. We selected 15 records for the literature review.

**Conclusion:**

Any patient who presents with trigeminal neuralgia which responds poorly to medical management should be properly examined for underlying primary systemic autoimmune diseases.

## INTRODUCTION

The neurological manifestations of connective tissue diseases (CTD) include meningitis, cerebellar ataxia, seizures, neuropsychosis, and transverse myelitis, cranial and peripheral neuropathy.^1^ These manifestations are the result of either vasculitis or vasculopathy due to secondary antiphospholipid syndrome. Pure trigeminal sensory neuropathy or trigeminal neuralgia (TN) is the most common central nervous system manifestation of mixed connective tissue disease (MCTD).^2^ It usually presents as sudden severe paroxysms of excruciating pain, tingling, and numbness on one side of the face which usually lasts a few seconds to a few minutes, involving one or more branches of the trigeminal nerve. However, there are few case reports regarding CTDs presenting as TN. Herein, we report four patients with various systemic autoimmune diseases where unilateral trigeminal sensory neuropathy was the initial manifestation.

## MATERIAL AND METHODS

**Figure 1.**
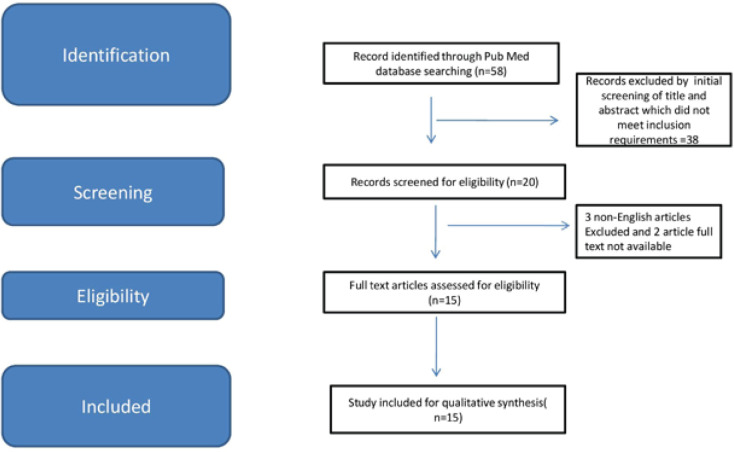
Flow diagram of the systemic search.

There are sparse evidence in literature on trigeminal neuralgia as an initial presentation of CTD. The relevant literature describing trigeminal neuralgia in CTD was reviewed. The authors performed a systematic search of patients with Trigeminal neuralgia and Connective tissue diseases in PubMed, Scopus from January 1970 until July 2022. Keywords in the search were “Trigeminal Neuralgia”[MeSH Terms] AND (“Connective tissue diseases”[MeSH Terms] OR “Systemic sclerosis”[All Fields] OR “Sjogren’s syndrome”[MeSH Terms] OR “Mixed connective tissue disease”[MeSH Terms]) OR “autoimmune diseases” [MeSH Terms]). The language of the chosen articles was restricted to English. The discussion was based on the case study and a literature review. After screening, we selected 15 records for review as shown in **Figure 1**.

## CASE 1

A 26-year-old female presented with complaints of continuous tingling and numbness in the nose, cheek, mouth, tongue, and chin on the right side for 2 months. There was no history of fever, chest pain, dyspnoea, skin rash, oral ulcer, arthralgia, and myalgia. Her past medical history was unremarkable. On detailed history it was noted that she had the paleness of fingers on cold exposure for the last 1 year. Physical examination revealed bilateral swollen hands and fingers with acrosclerosis. Nail fold capillaroscopy showed abnormal capillaries with an active scleroderma-like pattern. Neurological examination showed hypoesthesia in the maxillary and mandibular branches of the right trigeminal nerve with intact corneal reflex. The jaw jerk, motor component of the fifth nerve, and the remainder of the nervous system examination were normal. Systemic examination was unremarkable. Axial gadolinium-enhanced T1-weighted magnetic resonance imaging of the brain showed no contrast enhancement in the cisternal part of the right trigeminal nerve. Laboratory investigations revealed positive ANA (1:320 titre) with speckled pattern, anti-RNP antibody > 200.0 U/ml (normal <10U/ml) with negative anti-Ro/La, Sm, dsDNA and Scl-70. She had normal complement levels. The serum creatine phosphokinase (CPK) was 120 IU/mL (normal, < 145 IU/mL), the erythrocyte sedimentation rate (ESR) was 64 mm/h, and serum creatinine was 0.82 mg/dL. The blood cell count, platelet count, liver function test, and urine analysis were normal. Her serology for hepatitis B, hepatitis C, and HIV were negative and HRCT thorax was normal. Echocardiography was negative for pulmonary hypertension. Based on Raynaud’s phenomenon, swollen fingers, acrosclerosis, and positive serology for anti-RNP antibodies, she was diagnosed with MCTD according to Kasukawa’s criteria.^3^ She was started on prednisolone 20mg/day, tadalafil 10 mg/day with hydroxychloroquine 200 mg/day. After two months of treatment, her Raynaud’s phenomenon, swelling of the hands, positive sensory symptoms improved. However, she continued to have mild facial numbness. She received symptomatic therapy with gabapentin 300mg per day for facial numbness. She had good response to gabapentin.

## CASE 2

A 30-year-old female had presented with a history of left side facial numbness and tingling with dental pain of 6 months duration. She was treated with carbamazepine with partial improvement. She denied fever, cough, chest pain, arthritis, shortness of breath, myalgia. She had Raynaud’s phenomenon for the last 2 years. She complained of left foot paraesthesia followed by acute left foot drop of 1-week duration. Two weeks later, she again complained of tingling, numbness, and weakness of the right foot. There was no history of proximal weakness. She had no bladder, bowel, or other cranial nerve involvement. Physical examination revealed puffy hands with sclerodactyly. She had reduced sensory perception in the area supplied by maxillary and mandibular branches of the left trigeminal nerve with intact corneal reflex. Neurological examination revealed hypotonia in both her lower limbs. There was weakness affecting the dorsiflexors, invertors, and evertors of both feet. Power was Medical Research Council (MRC) grade 3/5 and 2/5 distally in her right and left lower limbs, respectively. All deep tendon jerks were normal. However, bilateral ankle reflex was absent. The sensation was decreased over the S1–S2 dermatomes. Vibration and proprioception sense was absent in the left leg and decreased on the right. Other system examinations were normal. Her electromyographic findings are consistent with axonal sensorimotor neuropathy. Right sural nerve biopsy confirmed vasculitic neuropathy. Laboratory investigations revealed positive ANA (Speckled pattern, 1:160) with anti-U1RNP antibody level 100.0 U/ml (normal <10U/ml) and negative anti-Ro/La, Sm, dsDNA and Scl-70, anti-neutrophilic cytoplasmic antibody (ANCA). Her complement levels were normal. Her routine blood test showed a total leucocyte count of 10,500/mm^3^, platelets 520,000/cmm, raised acute phase reactants like ESR 62 mm in 1st hour and C-reactive protein value 20 mg/L (Normal < 5mg/L). Liver function, renal function test and urinalysis were normal. Her Hepatitis B, hepatitis C, syphilis, and human immune deficiency virus (HIV) serology were negative. A contrast MRI of the brain ruled out secondary causes of TN. She fulfilled Kasukawa’s criteria^3^ for diagnosis of MCTD. She was initially treated with intravenous pulse methylprednisolone at a dose of 500 mg daily for 3 days, followed by oral prednisolone (1 mg/kg daily) along with monthly intravenous cyclophosphamide at a dose of 15 mg/kg every 4 weeks for 6 months. On a follow-up visit after 8 weeks, she had partial recovery of bilateral foot drop and almost complete recovery of sensory loss. She also recovered from her symptoms of TN.

## CASE 3

A 42-year-old female presented with complaints of breathlessness on exertion, cough with expectoration for the last 2 months. She also complained of tightening of the skin around the mouth, face and thickening of the skin on both hands of 1-year duration along with Raynaud’s phenomenon. She had a history of heartburn. There was no history of fever, chest pain, haemoptysis, and wheeze. Occupational exposures including chemicals, dust, and smoke were not observed in the present case. Past history revealed intermittent severe pricking pain on the right side of the face, triggered by chewing, and cold drinks for the last five years suggestive of TN. She was treated with carbamazepine 200 mg thrice daily with partial relief. On physical examination, she had salt and pepper pigmentation on her face and neck, mask-like face, telangiectasia over the cheeks and sclerodactyly. There was skin tightening on the face, neck, trunk, and extremity with a modified Rodnan total skin thickness score (mRSS) of 12. Cardiac auscultation was normal. Respiratory examination revealed bilateral end-inspiratory crackles of velcro type and coarse crepitations over the bilateral infrascapular area. Other system examinations were normal. Neurological examination showed hypoesthesia in the maxillary and mandibular branches of the right trigeminal nerve with intact corneal reflex. Laboratory investigations revealed normal haemogram, erythrocyte sedimentation rate [ESR], 20 mm/hour. She had normal renal and liver function tests. Immunological tests showed a high positive antinuclear antibodies (ANA) titre of 1:320 with a nucleolar pattern. She had positive anti-Scl 70 antibodies with negative anti-double-stranded DNA antibodies, negative antiphospholipid antibodies, and normal complement levels. Nail fold capillaries showed an advanced scleroderma pattern with capillaries loss. The pulmonary function test showed a restrictive pattern with low FVC (62%) with a reduced DLCO. A high-resolution computer tomogram (HRCT) thorax showed interstitial lung disease with a non-specific interstitial pneumonitis pattern. Her echocardiography was normal. MRI study of the brain was unremarkable. She was diagnosed with diffuse cutaneous systemic sclerosis (SSc-dc) by 2013 ACR/EULAR classification criteria. She was started on mycophenolate mofetil (MMF) 2 gm per day in divided doses along with tadalafil 10mg per day which resulted in improvement in her respiratory and skin symptoms, however, her TN symptoms persisted.

## CASE 4

A 35-year-old female presented with additive onset arthritis affecting both small and large joints with early morning stiffness for the last 2-month duration. She also had a history of Raynaud’s phenomenon for the last 6 years with dryness of mouth for the last 4 months. There was no history of fever, skin rash, and muscle weakness. History revealed a severe, sharp, piercing intermittent electric shock like pain on the right side of her face of 2-year duration which was triggered by talking, washing his face, eating, and brushing his teeth. She was diagnosed with TN and was taking carbamazepine. However, she neither showed a satisfactory response to carbamazepine nor to any antiepileptics/antidepressants. On examination, there was skin tightening of fingers and hand with a modified Rodnan total skin thickness score (mRSS) of 4. On musculoskeletal examination, there were tender, and swollen joints involving bilateral MCPs, PIPs, wrists, elbows and knees along with a limited range of motion of the affected joints. Oral examination showed dry buccal mucosa, with multiple dental caries. Neurological examination showed hypoesthesia in the maxillary and mandibular branches of the right trigeminal nerve with intact corneal reflex. Her other system examinations were normal. Her complete blood count showed anaemia of chronic disease (Haemoglobin 9.8 g/dL) and her renal and liver function tests were normal. Her ESR and CRP were 88 mm/hr (normal <20 mm/hr) and CRP of 39 mg/dL (normal <6 mg/dL) respectively. The rheumatoid factor and anti-CCP were negative. Plain radiographs of the hand showed features suggestive of inflammatory arthritis without erosions. Her hepatitis B, hepatitis C, and human immune deficiency virus (HIV) serology were negative. Her lung function tests and HRCT of the thorax were normal with normal echocardiography findings. Schirmer test on both eyes showed 3mm for the left eye wetting of the test strip after 5 min and 4mm for the right eye. Minor salivary gland biopsy showed signs of sialoadenitis comprised of lymphocytic infiltration with more than one lymphocytic focus (50 lymphocytes) per 4 mm^2^. Nail fold capillaroscopy showed an active scleroderma-like pattern. Immunological tests showed a high positive antinuclear antibodies (ANA) titre of 1:160 with speckled as well as centromere pattern. ELISA for anti-Centromere antibody, anti-Ro antibodies, and anti-La antibodies was positive. She was diagnosed with limited cutaneous systemic sclerosis (SSc-lc) based on the 2013 ACR/EULAR classification criteria. Clinical findings including the ocular symptoms of dry eyes and oral sicca symptoms, and positive autoantibody of anti-SSA (Ro) antibody were compatible with diagnostic criteria for Sjögren’s syndrome (American European Consensus Group 2002 revised classification criteria for SS). With a diagnosis of the overlap syndrome of primary Sjögren’s syndrome and limited systemic sclerosis (pSS-SSc), she was started on methotrexate, hydroxychloroquine, and low dose corticosteroid. Her joint symptoms improved however her sicca symptoms and TN persisted for which she received symptomatic therapy with gabapentin 300mg per day. She had good relief of pain with gabapentin.

## DISCUSSION

Trigeminal neuropathy or tic douloureux is usually seen in malignancy, trauma, connective tissue diseases, and infections (4). Isolated TN is found in many connective tissue disorders such as systemic lupus erythematosus (SLE), systemic sclerosis (SSc), and polymyositis/dermatomyositis (PM/DM), primary Sjögren′s syndrome, MCTD and rarely in rheumatoid arthritis. As per the available literature, trigeminal neuralgia can be the presenting feature of different autoimmune systemic diseases like MCTD, pSS and systemic sclerosis (**Table 1**). In our series, two patients had MCTD, one had diffuse SSc and one had Overlap syndrome (pSS-SSc); all of them had TN as initial clinical presentation.

**Table 1.** Various case studies regarding trigeminal neuralgia as an initial presentation of systemic autoimmune diseases.

**Study**	**Number of patients**	**Age**	**Sex**	**Laterality of TN**	**TN to CTD Diagnosis duration (months)**	**Diagnosis**	**Treatment**	**Improvement of TN symptoms**
Kabadi UM et al., 1977 (22)	1	41	F	U/L	60	CREST	C	Partial
						syndrome		
Bennett RM et al.,1978 (28)	1	34	F	U/L	12	MCTD	P	No
Searles et al., 1978 (20)	1	38	F	B/L	0	MCTD	P	Partial
Burke MJ et al., 1979^12^	3	63	F	U/L	24	SSc	Pencillamine	No
		22	M	U/L	1			
		36	M	U/L	24			
Teasdall RD et al., 1980^9^	5	43	M	U/L	24	SSc	NA	No
		51	F	U/L	60		NA	No
		55	F	U/L	18		NA	No
		52	F	U/L	12		NA	No
		63	F	U/L	12		NA	No
Edmondstone WM et al.,1982^5^	1	25	M	U/L	13	MCTD	P	Partial
Heald A et al., 1989^10^	1	58	M	B/L	72	SSc	Penicillamine, Naproxen	No
Varga et al.,1990^18^	1	39	M	B/L	NA	MCTD	P,A	Partial
Alfaro-Giner A et al., 1992^26^	1	41	F	U/L	2	MCTD	P/C	No
Hojaili B et al., 2006^4^	1	46	F	B/L	2	MCTD	C/G/P/A	No
Ribeiro RT et al., 2009^8^	2	42	M	U/L	12	SSc	G/N/M/P	Partial
		50	F	U/L	10		A/P/M	No
Hamoir et al., 2014^24^	1	59	F	U/L	0	MCTD	P/HCQ	NA
Danve et al., 2018^25^	1	55	F	B/L	0	MCTD	P/IV IG	Partial
Yuan J et al., 2018^15^	1	30	F	U/L	0	pSS	P/C	Yes
Jeemon G et al., 2022^27^	1	32	F	U/L	4	MCTD	C	Yes

C: Carbamazepine; G: Gabapentin; P: Prednisolone; A: Azathioprine; M: Methotrexate; N: Nortriptyline; IV IG: Intravenous immunoglobulin; HCQ: Hydroxychloroquine; TN: Trigeminal Neuralgia; MCTD: Mixed connective tissue; pSS: Primary Sjögren’s syndrome; SSc: Systemic sclerosis; U/L: Unilateral; B/L: Bilateral; NA: Not available.

According to literature, approximately 10% to 17% of patients with MCTD have neuropsychiatric dysfunctions such as trigeminal neuritis, headache, aseptic meningitis, seizure, peripheral neuritis, cerebrovascular disease, and psychosis.^1,5^ Trigeminal neuropathy is the most common neurologic presentation in MCTD.^1,2,4^ Among the connective tissue diseases, MCTD may first manifest as acute, sensory predominant, and anatomically variable motor-sparing trigeminal neuropathy. This neuropathy seems to be, unilateral or bilateral, prodromal or late, occurring with or without pain, and having a variable prognosis.^6^ In isolated TN, the symptoms are bilateral in less than 4% of the cases.^7^ However, most MCTD cases reported in the literature were bilateral.^4^ All of our patients presented with unilateral TN. The facial pain described in MCTD is usually continuous, whereas, in idiopathic TN, the pain is paroxysmal.^4^

The majority of patients with SSc showed bilateral TN.^8–10^ The second and third divisions of the nerve are involved together in the majority of SSc patients with TN (∼80%). M.J Burkhe et al. (1979) described three cases of systemic sclerosis in which trigeminal neuropathy was the presenting symptom. In a study, trigeminal neuropathy has been identified in 16 (4 %) of 442 consecutive patients with progressive systemic sclerosis (PSS) who were first evaluated during the period between 1972 and 1980.^11^ TN could be a consequence of active inflammation (combination of micro-angiopathy and fibrosis) and represent a sign of disease activity in SSc.^9,12^

Similarly, neurologic manifestations occur in 20% to 25% of patients with primary Sjögren syndrome (pSS), may precede sicca symptoms in 33–93% of patients.^13–16^ Bilateral involvement appears to be more common in pSS compared with idiopathic TN.^17^ Trigeminal nerve dysfunction in SS is caused by damage to the gasserian ganglion, thus sparing the ophthalmic division and thereby preserving the corneal reflex. In a Japanese study trigeminal neuropathy was found to be the most frequent neurological symptom in 21 female patients with primary SS; it occurred in eight (50%) of the 16 patients with objective neurological abnormalities.^17^ Our 4th case with pSS-SSc overlap syndrome had TN which preceded sicca symptoms.

Although, vascular compression is the most prevalent cause in idiopathic TN, normal brain imaging ruled out neurovascular compression in our all cases. The pathophysiology of trigeminal neuropathy in CTD is still unknown. Immune complex formation in the sensory nerve root or ischemia of vasa nervosum due to vasculitis and demyelination is likely the cause of TN in CTDs.^4,18,19^ Vasculitis affects the trigeminal nerve branches, particularly where they are tightly invested by dura and are more prone to oedema, as well as an increase in endoneurial pressure as a consequence. Fibrosis of the epineurium and perineurium might indeed increase endoneurial pressure and damage myelinated trigeminal fibres selectively. Direct injury caused by vasculopathy may affect the blood-brain barrier, allowing antibodies to enter the central nervous system. There is experimental evidence that the trigeminal ganglion blood vessels are more permeable to proteins and antibodies than the blood-brain barrier.^19,20^ Vasculitic changes, neural tissue fibrosis, and increased permeability of trigeminal blood vessels are the possible mechanisms in trigeminal neuralgia.

In a retrospective study involving 81 patients having CTD with TN, 21 patients (26%) and 15 (19%) had MCTD and SSc respectively. The neuropathy developed before the symptoms of CTD in 6/81 patients (7%), and in 38/81 patients (47%) TN and CTD were diagnosed concurrently. 12% had mild improvement and 3% had marked improvement of numbness in 1 year follow up; no patient had a complete return of sensation.^2^

There is no effective curative therapy for trigeminal neuropathy (21); anti-epileptics and antidepressants may provide some relief.^8,15,22,27^ Corticosteroid therapy, intravenous immunoglobulin, or immunosuppressive treatments used in CTD are often ineffective in trigeminal sensory neuropathy.^19,23–28^

This case series highlights that trigeminal neuropathy may rarely be the presenting manifestation of systemic autoimmune diseases. A careful history and detailed clinical examination are helpful in establishing the correct diagnosis.
